# Chrysin Attenuates Cell Viability of Human Colorectal Cancer Cells through Autophagy Induction Unlike 5-Fluorouracil/Oxaliplatin

**DOI:** 10.3390/ijms19061763

**Published:** 2018-06-14

**Authors:** Yueh-Ming Lin, Chih-I Chen, Yi-Ping Hsiang, Yung-Chia Hsu, Kung-Chuan Cheng, Pei-Hsuan Chien, Hsiao-Lin Pan, Chien-Chang Lu, Yun-Ju Chen

**Affiliations:** 1 Division of Colorectal Surgery, Department of Surgery, Kaohsiung Chang Gung Memorial Hospital and Chang Gung University College of Medicine, Kaohsiung 833, Taiwan; porta2005@gmail.com (Y.-M.L.); topguncheng@gmail.com (K.-C.C.); 2 Division of Colon and Rectal Surgery, Department of Surgery, E-Da Hospital, Kaohsiung 824, Taiwan; jimmyee0901@gmail.com; 3 School of Medicine for International Students, I-Shou University, Kaohsiung 824, Taiwan; 4 Department of Pharmacy, E-Da Hospital, Kaohsiung 824, Taiwan; ed108228@edah.org.tw; 5 Department of Pharmacy, E-Da Cancer Hospital, Kaohsiung 824, Taiwan; ed108840@edah.org.tw; 6 Department of Medical Research, E-Da Hospital, Kaohsiung 824, Taiwan; peihsuan68@gmail.com (P.-H.C.); dkiyh@yahoo.com.tw (H.-L.P.)

**Keywords:** CRC, chrysin, chemotherapy, autophagy, ROS

## Abstract

Chemotherapeutic 5-fluorouracil (5-FU) combined with oxaliplatin is often used as the standard treatment for colorectal cancer (CRC). The disturbing side effects and drug resistance commonly observed in chemotherapy motivate us to develop alternative optimal therapeutic options for CRC treatment. Chrysin, a natural and biologically active flavonoid abundant in propolis, is reported to have antitumor effects on a few CRCs. However, whether and how chrysin achieves similar effectiveness to the 5-FU combination is not clear. In this study, 3-(4,5-dimethylthiazol-2-yl)-2,5-diphenyltetrazolium bromide (MTT), western blotting, fluorescence microscopy, and reactive oxygen species (ROS) production were assayed. We found that chrysin exhibited similar inhibition of cell viability as the 5-FU combination in a panel of human CRC cells. Furthermore, the results showed that chrysin significantly increased the levels of LC3-II, an autophagy-related marker, in CRC cells, which was not observed with the 5-FU combination. More importantly, blockage of autophagy induction restored chrysin-attenuated CRC cell viability. Further mechanistic analysis revealed that chrysin, not the 5-FU combination, induced ROS generation, and in turn, inhibited the phosphorylation of protein kinase B (Akt) and mammalian target of rapamycin (mTOR). Collectively, these results imply that chrysin may be a potential replacement for the 5-FU and oxaliplatin combination to achieve antitumor activity through autophagy for CRC treatment in the future.

## 1. Introduction

Colorectal cancer (CRC) is one of the most common cancer types in the world, and its occurrence is still on the rise yearly. Since CRC is a heterogeneous disease, both molecular expression patterns and pathological features reflect prognostic outcomes and treatment responses [1,2,3]. Although early-stage CRC has a better prognosis than other cancer types, it is frequently found that patients are diagnosed with advanced CRC because they had no obvious symptoms to start with. Currently, chemotherapy is often applied as the first-line treatment option for advanced CRC. A substantial improvement in survival for advanced CRC patients treated with chemotherapy was observed in the last two decades. Treatment options expanded from 5-fluorouracil (5-FU) only to regimens that combine 5-FU with oxaliplatin or irinotecan. To date, 5-FU combined with oxaliplatin is used most frequently as the standard treatment for advanced CRC [4,5,6]. Although it shows promising therapeutic effectiveness in CRC patients, disturbing side effects and drug resistance, which are commonly observed in chemotherapy, are hurdles for its application in cancer treatment [7,8,9,10,11]. This motivates us to develop alternative optimal therapeutic options.

Chrysin (5,7-dihydroxyflavone) is a naturally occurring and biologically active flavonoid found abundantly in propolis. It is frequently used as herbal medicine in some areas of Asia [12,13]. Accumulated studies demonstrated that chrysin has multiple functions. For example, it is reported to have potent anti-inflammatory activity. The underlying mechanisms may involve cyclooxygenase-2 (COX-2) or peroxisome proliferator-activated receptor-γ (PPARγ)-regulated M1/M2 status [14,15]. As a result, it can also be used as a health supplement. Furthermore, it displays promising antitumor activity in many cancer types. Results showed that it inhibits tumor growth via the induction of apoptosis, cell cycle arrest, and so on. Most importantly, no undesirable side effects or toxicity were reported [15,16,17]. Although chrysin is reported to have antitumor activity, whether it achieves similar effectiveness in CRC when compared to 5-FU combined with oxaliplatin is not yet known. Moreover, the underlying mechanism is not clarified in detail. In this study, we investigated and compared the therapeutic effectiveness and underlying mechanisms of chrysin with a combination of 5-FU and oxaliplatin in CRC. 

## 2. Results

### 2.1. Chrysin Achieved Similar Effectiveness to 5-Fluorouracil (5-FU) Combined with Oxaliplatin in a Panel of Colorectal Cancer (CRC) Cells

**Figure 1:**
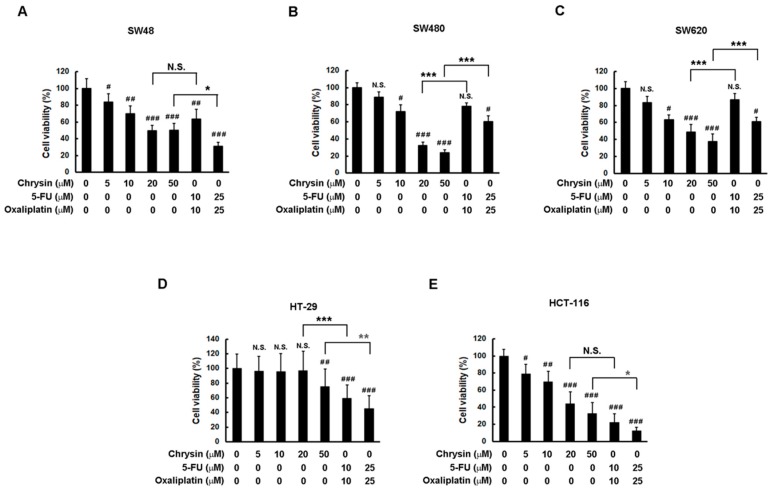
Chrysin achieved similar effectiveness to 5-fluorouracil (5-FU) combined with oxaliplatin in a panel of colorectal cancer (CRC) cells. (**A**–**E**) Each CRC cell line was treated with the indicated concentrations of chrysin or 5-FU and oxaliplatin for three days. Cell viability was determined via a 3-(4,5-dimethylthiazol-2-yl)-2,5-diphenyltetrazolium bromide (MTT) assay, and was statistically quantified. ^#^ *p* < 0.05; ^##^ *p* < 0.01; ^###^ *p* < 0.001 when compared with control (vehicle only) groups. * *p* < 0.05; ** *p* < 0.01; *** *p* < 0.001 when compared with indicated control groups. A *p*-value < 0.05 was considered as statistically significant. N.S.—not significant.

Firstly, a comparison of therapeutic effectiveness on cell viability between chrysin and 5-FU combined with oxaliplatin was performed through application to a panel of CRC cells. To ensure consistent comparisons, the concentration of chrysin was made equal to the sum of 5-FU and oxaliplatin concentrations. Then, CRC cells were treated with the indicated concentration of each drug for three days, and cell viability was determined via a 3-(4,5-dimethylthiazol-2-yl)-2,5-diphenyltetrazolium bromide (MTT) assay. As shown in Figure 1, chrysin indeed significantly attenuated cell viability in a dose-dependent manner in CRC cell lines when compared with the control (vehicle only) group. Although chrysin had a minor effect on HT-29 cells, its effect on other CRC cell lines was dramatic. When comparing the effect between chrysin and 5-FU combined with oxaliplatin, we found that chrysin achieved similar effectiveness in these CRC cell lines. Although chrysin had a better effect than 5-FU combined with oxaliplatin in some cell lines (Figure 1B,C) and a worse effect in others (Figure 1D,E), the therapeutic effectiveness on the attenuation of CRC cell viability was overall equivalent between chrysin and 5-FU combined with oxaliplatin.

### 2.2. LC3-II Levels Were Induced in Chrysin-Treated but Not 5-Fluorouracil (5-FU) /Oxaliplatin-Treated Colorectal Cancer (CRC) Cells

**Figure 2:**
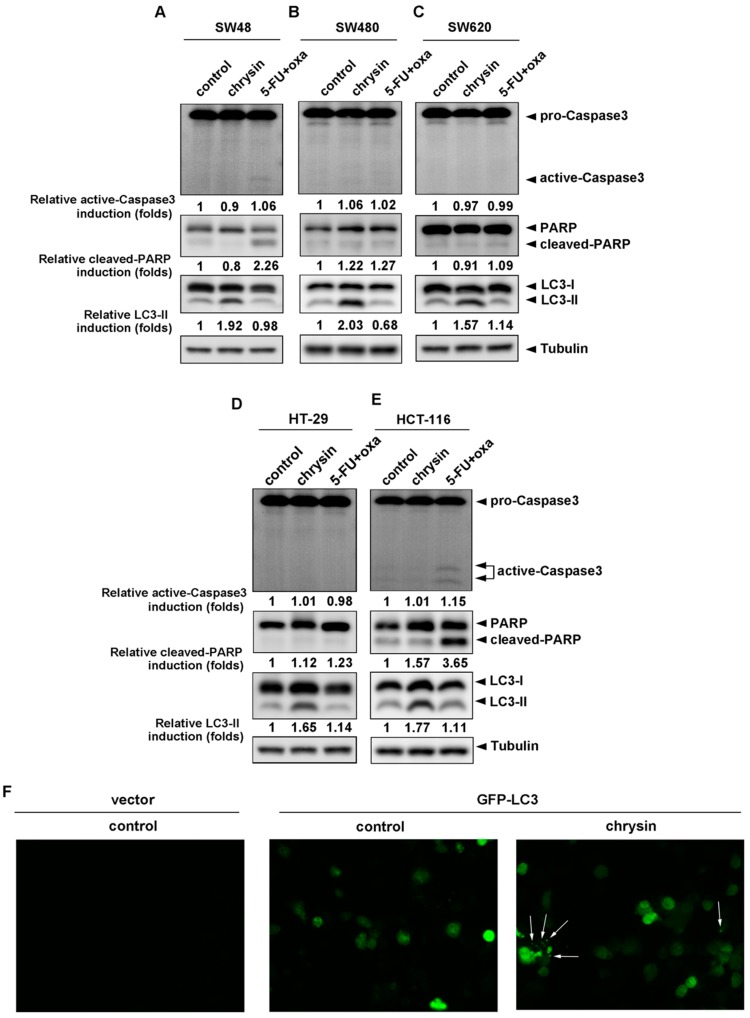
LC3-II levels were induced in chrysin-treated, but not 5-fluorouracil (5-FU)/oxaliplatin-treated colorectal cancer (CRC) cells. (**A**–**E**) Each CRC cell line was treated with the indicated concentrations of 50 μM chrysin or 25 μM 5-FU/25 μM oxaliplatin for 24 h. Then, cells were harvested for western blot analysis. The expressions of cleaved forms of caspase-3 and poly-ADP-ribose polymerase (PARP), as well as of LC3-II, were examined using indicated antibodies. The band images were quantified using tubulin as a calibrated control. Relative fold inductions of activated caspase-3, cleaved PARP, and LC3-II were indicated as compared to control groups. (**F**) The GFP-LC3 expression vector was transfected into HCT-116 cells, followed by treatment with 50 μM chrysin for 24 h. GFP-LC3 punctate structures were observed under a fluorescence microscope. White arrow represents GFP-LC3 punctate structure. The fluorescent images were displayed in a 10× multiplication.

Since chrysin achieved similar effectiveness in the attenuation of CRC cell viability when compared with 5-FU combined with oxaliplatin, we further analyzed how CRC cell viability was attenuated between these two groups. We examined the death pathways, apoptosis and autophagy, by detecting the expression of respective biomarkers—active caspase-3/cleaved poly-ADP-ribose polymerase (cleaved-PARP) for apoptosis and LC3-II for autophagy. As shown in Figure 2A–E, chrysin and the 5-FU combination were not much different with regards to the induction of apoptosis (upper panel) in most CRC cell lines. However, it is interesting that chrysin significantly increased LC3-II levels in all applied CRC cell lines, which was not observed in the 5-FU/oxaliplatin−treated group (lower panel). We further enforced HCT-116 cells expressing GFP-LC3, followed by chrysin treatment. During autophagy, LC3-II relocalizes to the autophagosomal membranes. Therefore, the accumulation of GFP-LC3 punctate structures provides a way of detecting autophagosomes. The results from fluorescence imaging analysis (Figure 2F) showed that GFP-LC3 punctate structures were not obviously detected in the control group (middle panel), but exhibited a significant increase in the chrysin-treated group (right panel). Collectively, this implies the involvement of different mechanisms underlying the antitumor activities of chrysin and 5-FU/oxaliplatin.

### 2.3. Blockage of Autophagy Induction Restored Chrysin-Attenuated Colorectal Cancer (CRC) Cell Viability

**Figure 3:**
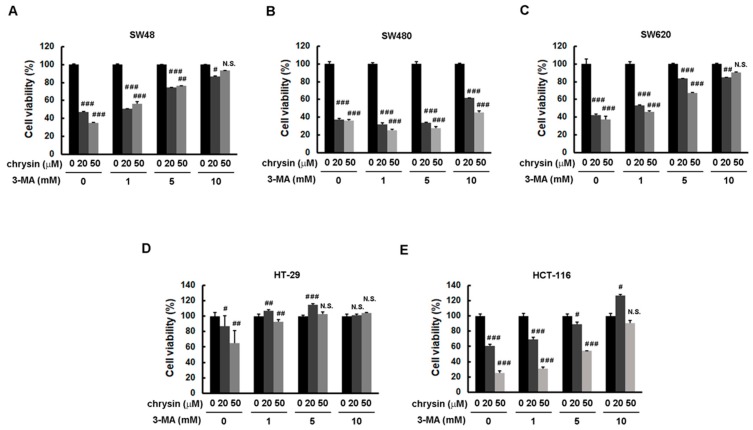
Blockage of autophagy induction reversed chrysin-attenuated colorectal cancer (CRC) cell viability. (**A**–**E**) Each CRC cell line was pretreated with the indicated concentration of 3-methyladenine (3-MA) followed by the indicated concentration of chrysin for three days. Cell viability was determined via a 3-(4,5-dimethylthiazol-2-yl)-2,5-diphenyltetrazolium bromide (MTT) assay, and was statistically quantified. ^#^ *p* < 0.05; ^##^ *p* < 0.01; ^###^ *p* < 0.001 when compared with indicated control groups. A *p*-value < 0.05 was considered as statistically significant. N.S.—not significant.

**Figure 4:**
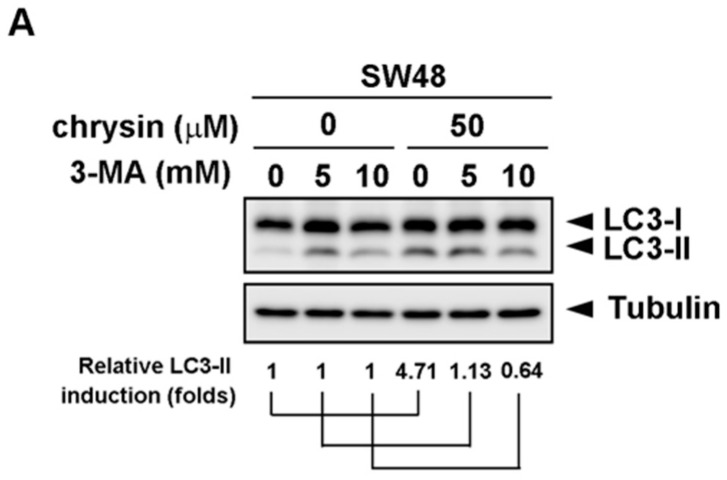

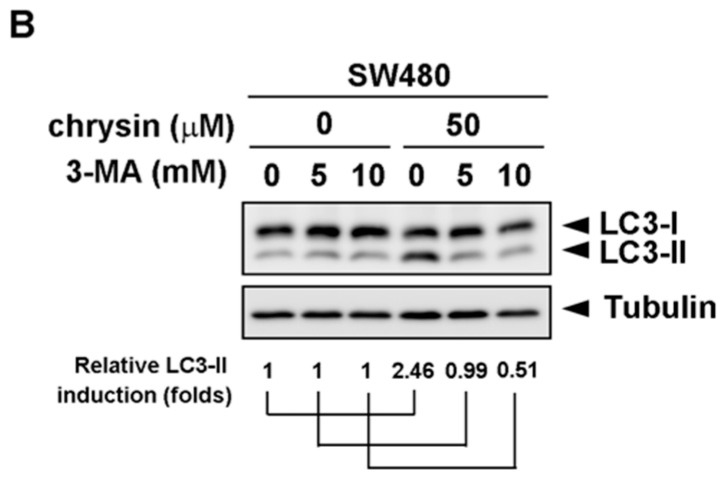
Autophagy inhibitor, 3-methyladenine (3-MA), reversed chrysin-induced LC3-II expression. (**A**,**B**) Colorectal cancer (CRC) cell lines were pretreated with the indicated concentration of 3-MA, followed by chrysin treatment for 24 h. Then, cells were harvested for western blot analysis. Expression of LC3-II was examined using indicated antibodies. The band images were quantified using tubulin as a calibrated control. Relative fold induction of LC3-II was indicated as compared to indicated control groups.

To further confirm the significance of chrysin-induced LC3-II production, the autophagy inhibitor, 3-methyladenine (3-MA), was applied. CRC cells were pretreated with the indicated concentration of 3-MA, followed by treatment with chrysin for three days. Cell viability was then determined via a 3-(4,5-dimethylthiazol-2-yl)-2,5-diphenyltetrazolium bromide (MTT) assay. As shown in Figure 3, treatment with chrysin alone attenuated cell viability in a dose-dependent manner. However, this dose-dependent inhibition was reversed when these CRC cells were pretreated with 3-MA. The degree of reversal was gradually enhanced with an increase in 3-MA concentration. Supporting these results, treatment with chrysin alone induced LC3-II expression, whereas such induction was not observed in cells treated with 3-MA (Figure 4). These results suggest that chrysin attenuates CRC cell viability at least in part through autophagy induction. 

### 2.4. Chrysin-Mediated Autophagy Induction Was through Protein Kinase B (Akt)/Mammalian Target of Rapamycin (mTOR) Signaling Pathway in Colorectal Cancer (CRC) Cells

**Figure 5:**
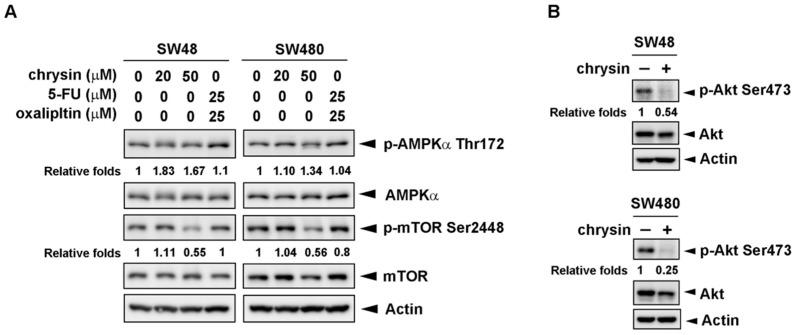
The protein kinase B (Akt)/mammalian target of rapamycin (mTOR) signaling pathway was inhibited in chrysin-treated but not 5-fluorouracil (5-FU)/oxaliplatin-treated colorectal cancer (CRC) cells. (**A**,**B**) SW48 and SW480 cells were treated with the indicated concentrations of chrysin or 5-FU/oxaliplatin for 24 h. Then, cells were harvested for western blot analysis. Expressions of phosphorylated AMP-activated protein kinase alpha (p-AMPKα) threonine 172, AMPKα, phosphorylated mTOR (p-mTOR) serine 2448, mTOR, phosphorylated Akt (p-Akt) serine 473, and Akt were examined using the indicated antibodies. The band images were quantified using both actin and the AMPKα, mTOR, or Akt proteins as calibrated controls. Relative fold inductions of p-AMPKα threonine 172, p-mTOR serine 2448, and p-Akt serine 473 were indicated as compared to control (vehicle alone) groups.

Next, we investigated the mechanism underlying chrysin-mediated autophagy induction. AMP-activated protein kinase (AMPK) is a well-known energy sensor and upstream regulator of autophagy. Under starvation conditions, AMPK is activated and, in turn, inhibits mammalian target of rapamycin 1 (mTOR1) activity, followed by decreasing protein synthesis and increasing autophagy [18,19]. We first examined the activation of AMPK and mTOR. As shown in Figure 5A, chrysin significantly inhibited mTOR phosphorylation in CRC cells, which is expected to induce autophagy. AMPK phosphorylation was increased by chrysin. In addition to AMPK, Akt is reported as another upstream regulator of autophagy through the targeting of mTOR [20,21]. The results in Figure 5B show that chrysin treatment obviously attenuated Akt phosphorylation in CRC cells. These results suggest that chrysin inhibits Akt activation and, in turn, decreases mTOR phosphorylation, which eventually leads to autophagy induction in CRC cells.

### 2.5. Chrysin Induced the Production of Reactive Oxygen Species (ROS) in Colorectal Cancer (CRC) Cells

**Figure 6:**
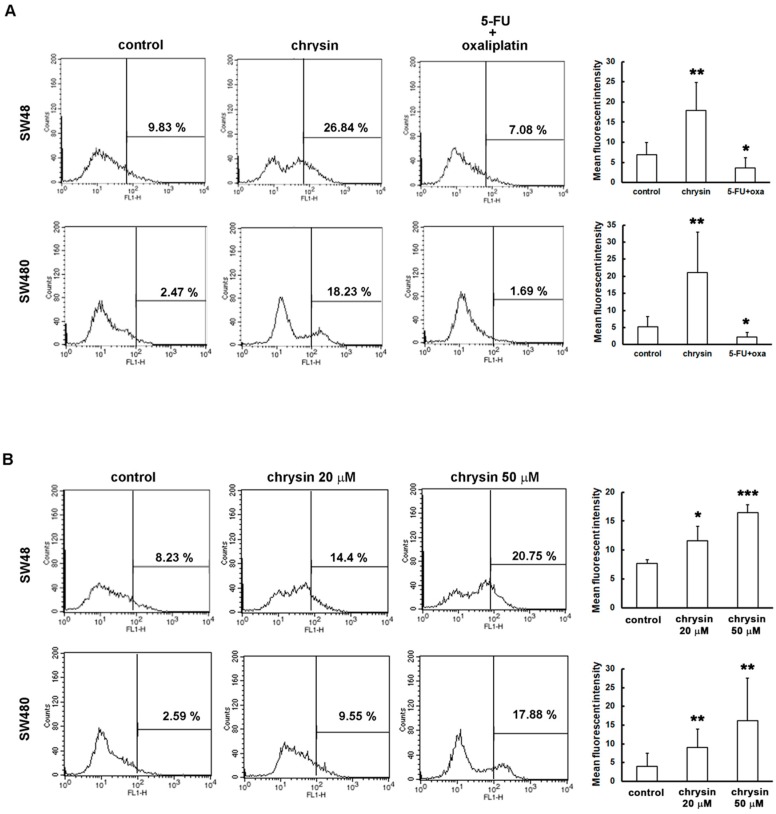
Chrysin, not 5-fluorouracil (5-FU)/oxaliplatin, induced the production of reactive oxygen species (ROS) in colorectal cancer (CRC) cells. (**A**,**B**) SW48 and SW480 cells were seeded overnight, and treated with the indicated concentrations of 50 μM chrysin or 25 μM 5-FU/25 μM oxaliplatin for 24 h. Then, following trypsinization, cell pellets were stained with 10 μM CM-H_2_DCFDA at 37 °C in the dark for 30 min. Intracellular ROS level was measured using flow cytometry, and was statistically quantified. * *p* < 0.05; ** *p* < 0.01; *** *p* < 0.001 when compared with indicated control groups. A *p*-value < 0.05 was considered as statistically significant.

Recently, several studies indicated that ROS induce autophagy through Akt/mTOR inhibition [22,23,24]. Notably, chrysin was reported to induce cell death through ROS production in prostate cancer and chronic lymphocytic leukemia [25,26]. Whether chrysin induces ROS production in CRC cells is not yet known. We further elucidated this issue. ROS production was analyzed using CM-H_2_DCFDA staining and flow cytometry. As shown in Figure 6A, ROS production was significantly increased in chrysin-treated CRC cells. Interestingly, such an effect was not observed in CRC cells treated with 5-fluorouracil (5-FU)/oxaliplatin. Furthermore, the level of ROS production was gradually enhanced with an increase in chrysin concentration (Figure 6B). Taken together, these results suggest that chrysin induces ROS production and, in turn, leads to autophagy through Akt/mTOR inhibition, unlike the effect of 5-FU/oxaliplatin.

## 3. Discussion

Colorectal cancer (CRC) is a cancer with a better prognosis than other cancer types. However, when CRC tumors progress to the advanced stage, the survival rate drops quickly. Currently, chemotherapy seems to be the only effective treatment option. Although chemotherapy is effective, the serious side effects often force patients to withdraw from treatment, and seek a better quality of life. Even when patients continue chemotherapy, acquired resistance eventually develops. These two problems severely challenge the clinical application of chemotherapy [27]. Developing alternative treatment options with similar therapeutic effectiveness but no undesirable adverse effects seems necessary. In this study, we found that a natural product, chrysin, induces similar effects on cell viability inhibition in a panel of human CRC cells when compared with a combination of 5-fluorouracil (5-FU) and oxaliplatin. Although chrysin is reported to have antitumor activity, it is not known whether it has similar effectiveness as chemotherapy in CRC treatment. Our study is the first to investigate this issue. Since chrysin was demonstrated to be harmless to normal cells, and to have multiple functions including anti-inflammation and anti–cancer progression, it is usually claimed as a health supplement to prevent disease [28,29]. Based on these studies and our results, chrysin may have significant potential for CRC treatment in the future. For example, the doses of 5-FU/oxaliplatin may be reduced to attenuate the degree of side effects by combining chrysin treatment. Alternatively, chrysin was reported as a potent inhibitor of breast cancer resistance protein (BCRP), which is one of the ATP-binding cassette (ABC) transporters, and commonly involved in the multidrug resistance (MDR) of chemotherapy [30]. This implies that chrysin may also have potential for use in interval or combination therapy to prevent the occurrence of resistance commonly observed in chemotherapy, a feature which deserves investigation. Although chrysin has potential for clinical application, as supported by in vivo results showing that chrysin efficiently attenuated tumor growth in a xenograft mouse model of several cancer types [31,32,33], chrysin was intravenously administered to mice in these studies. Chrysin was reported as having low oral bioavailability, mainly due to extensive metabolism and the efflux of metabolites back into the intestine for hydrolysis and fecal elimination [34]. Thus, improving the poor bioavailability is the first priority to address before considering clinical application of chrysin.

In the mechanistic analysis of the inhibition of CRC cell viability, we found that chrysin, not the 5-FU/oxaliplatin combination, induced reactive oxygen species (ROS) production and, in turn, led to autophagy induction, as detected by an increase in LC3-II levels, through protein kinase B (Akt)/mammalian target of rapamycin (mTOR) inhibition. This is why blockage of autophagy induction restored chrysin-attenuated CRC cell viability. This is the first time that chrysin is reported to induce ROS production and autophagy in CRC cells. Since autophagy is one mode of programmed cell death [35], our results imply that chrysin attenuates cell viability at least in part through autophagy-dependent cell death. Notably, our findings seem to conflict with studies stating that chrysin could protect rat kidneys from paracetamol-induced autophagy, and sensitize human glioblastoma cells to temozolomide by downregulating autophagy [36,37]. However, it is well known that whether autophagy induces cell death or survival depends on the cancer type and tumor microenvironment [38]. Moreover, some natural compounds from herbs are reported to potentially function as autophagy inducers for cancer therapy [39]. Therefore, the exact role of chrysin in autophagy-mediated CRC cell death awaits further investigation.

## 4. Materials and Methods

### 4.1. Cell Lines and Reagents

SW48, SW480, and SW620 colorectal cancer (CRC) cells were maintained in L15 medium; HT-29 and HCT-116 CRC cells were maintained in McCoy’s 5A medium. The medium for the CRC cells was also supplemented with 10% fetal bovine serum (Logan, UT, USA). Chrysin, 3-methyladenine (3-MA), and the antibody against tubulin were purchased from Sigma-Aldrich (St. Louis, MO, USA). The antibodies against poly-ADP-ribose polymerase (PARP), phosphorylated protein kinase B (Akt) (p-Akt) serine 473, Akt, phosphorylated AMP-activated protein kinase alpha (AMPKα) (p-AMPKα) threonine 172, AMPKα, phosphorylated mammalian target of rapamycin (mTOR) (p-mTOR) serine 2448, and mTOR were obtained from Cell Signaling (Danvers, MA, USA). The antibody against caspase-3 was purchased from BioVision (Milpitas, CA, USA). The antibody against LC-3 was obtained from Novus (Littleton, CO, USA). The CM-H2DCFDA dye was purchased from Thermo Fisher Scientific (Waltham, MA, USA). Chrysin, and the combined 5-fluorouracil (5-FU) and oxaliplatin were dissolved in dimethylsulfoxide (DMSO), and the stock solution was prepared as 50 mM. 3-MA was dissolved in 95% ethanol with heating, and working solutions were freshly prepared prior to use. CM-H2DCFDA dye was reconstituted only as required by using DMSO, and working solutions were freshly prepared prior to use.

### 4.2. Cell Viability Assay

Cell viability was assessed using 3-(4,5-dimethylthiazol-2-yl)-2,5-diphenyltetrazolium bromide (MTT) colorimetric assays. Cells at a density of 5 × 10^3^ cells/well were seeded on 96-well plates overnight, and then treated with the indicated drugs. Three days later, relative cell amounts were determined by adding 1 μg/mL MTT to each well. Then, the medium was removed after 4 h incubation, and formazan was solubilized in 100 μL DMSO per well, followed by the measurement of absorbance at 570 nm.

### 4.3. Immunofluorescence Staining

The GFP-LC3 expression vector was transfected into HCT-116 cells at a ratio of 1:3 DNA to T-pro transfection reagent for two days, followed by treatment with 50 μM chrysin. Then, cells were fixed with 4% paraformaldehyde in phosphate buffered saline (PBS), followed by permeabilization using 1% Triton X-100 in PBS. The GFP-LC3 punctate structures were observed under a fluorescence microscope.

### 4.4. Detection of Intracellular Reactive Oxygen Species (ROS)

SW48 and SW480 cells were seeded overnight, and treated with the indicated concentration of chrysin or 5-fluorouracil (5-FU)/oxaliplatin for 24 h. Then, cells were trypsinized and centrifuged. The cell pellets were stained with 10 μM CM-H_2_DCFDA at 37 °C in the dark for 30 min. Intracellular ROS levels were measured via flow cytometry.

### 4.5. Statistical Analysis

The statistical analysis was performed using a Student’s *t*-test. *^/#^ *p* < 0.05, **^/##^ *p* < 0.01, and ***^/###^ *p* < 0.001 when compared with indicated control groups. A *p*-value < 0.05 was considered as statistically significant. N.S. means not significant.

## Abbreviations

5-FU	5-fluorouracil
CRC	colorectal cancer
COX-2	cyclooxygenase-2
PPARγ	peroxisome proliferator-activated receptor-γ
cleaved-PARP	cleaved poly-ADP-ribose polymerase
3-MA	3-methyladenine
ROS	reactive oxygen species
BCRP	breast cancer resistance protein
ABC	ATP-binding cassette
MDR	multidrug resistance
PBS	phosphate buffered saline
MTT	3-(4,5-dimethylthiazol-2-yl)-2,5-diphenyltetrazolium bromide
AMPKα	AMP-activated protein kinase alpha
mTOR	mammalian target of rapamycin
